# Antioxidative Properties of Fermented Soymilk Using *Lactiplantibacillus plantarum* LP95

**DOI:** 10.3390/antiox12071442

**Published:** 2023-07-18

**Authors:** Francesco Letizia, Alessandra Fratianni, Martina Cofelice, Bruno Testa, Gianluca Albanese, Catello Di Martino, Gianfranco Panfili, Francesco Lopez, Massimo Iorizzo

**Affiliations:** Department of Agriculture, Environmental and Food Sciences, University of Molise, Via De Sanctis, 86100 Campobasso, Italy; f.letizia@studenti.unimol.it (F.L.); fratianni@unimol.it (A.F.); martina.cofelice@unimol.it (M.C.); bruno.testa@unimol.it (B.T.); g.albanese@studenti.unimol.it (G.A.); panfili@unimol.it (G.P.); lopez@unimol.it (F.L.);

**Keywords:** functional food, soymilk, fermentation, isoflavones, *Lactiplantibacillus plantarum*

## Abstract

In recent times, there has been a growing consumer interest in replacing animal foods with alternative plant-based products. Starting from this assumption, for its functional properties, soymilk fermented with lactic acid bacteria is gaining an important position in the food industry. In the present study, soymilk was fermented with *Lactiplantibacillus plantarum* LP95 at 37 °C, without the use of stabilizers as well as thickeners and acidity regulators. We evaluated the antioxidant capacity of fermented soymilk along with its enrichment in aglycone isoflavones. The conversion of isoflavone glucosides to aglycones (genistein, glycitein, and daidzein) was analyzed together with antioxidant activity (ABTS) measurements, lipid peroxidation measurements obtained by a thiobarbituric acid reactive substance (TBARS) assay, and apparent viscosity measurements. From these investigations, soymilk fermentation using *Lp. plantarum* LP95 as a starter significantly increased isoflavones’ transformation to their aglycone forms. The content of daidzein, glycitein, and genistein increased after 24 h of fermentation, reaching levels of 48.45 ± 1.30, 5.10 ± 0.16, and 56.35 ± 1.02 μmol/100 g of dry weight, respectively. Furthermore, the antioxidant activity increased after 6 h with a reduction in MDA (malondialdehyde). The apparent viscosity was found to increase after 24 h of fermentation, while it slightly decreased, starting from 21 days of storage. Based on this evidence, *Lp. plantarum* LP95 appears to be a promising candidate as a starter for fermented soymilk production.

## 1. Introduction

The growing interest of consumers in nutritionally enriched and health-promoting foods provokes interest in the eventual development of fermented functional foods [1]. In this view, the consumption of soybean (*Glycine max*) and its products has received attention from researchers and consumers both for the nutritional value and for its potential health benefits, including reducing the risk of cardiovascular diseases, diabetes, breast cancer, and chronic inflammation, due to the presence of bioactive compounds, such as isoflavones [2,3]. The water extract of soybeans, namely, soymilk, is an important oriental traditional beverage [4], considered a colloidal system as it contains protein and minerals [5]. This beverage can be a good substitute for animal-based milk and an ideal food for vegans diets and lactose-intolerant people [6]. Despite the listed properties of soymilk, its consumption in Western countries is still limited due to its unpleasant taste and its antinutritional factor, such as phytates that compromise the natural availability of minerals, as well as the presence of indigestible oligosaccharides, such as raffinose and stachyose, that cause flatulence [7,8]. Considering these limitations, the fermentative biotransformation approach using lactic acid bacteria (LAB) is an effective strategy to improve the nutritional, textural, and rheological properties of soymilk [3]. Isoflavones are phytochemical compounds naturally present in soymilk, and their enteric absorption is related to their chemical structure. The concentrations of the different forms of these bioactive compounds in soymilk are related to the soybean cultivar, time of soaking, the ratio of soybean to water, filtration, and thermal treatments [9,10]. The principal isoflavones of soy are daidzein, genistein, and glycitein, which exist in plants conjugated with sugars [11,12]. 

Glycosides can be hydrolyzed in the oral cavity [13] and, mainly, in the intestine to their aglycones, which are then absorbed in the intestine [14]. The flavonoid glycosides are considered biologically inactive, and their bioavailability requires the initial hydrolysis of the sugar moiety by intestinal β-glucosidases for uptake into the peripheral circulation [15].

Several studies have shown that it is possible to reverse the glucoside/aglycone ratio in soy food through LAB strains with β-glucosidase activity [16,17]. Therefore, the use of these bacteria as a starter, with the aforementioned enzymatic activity, in the fermentation of soymilk could contribute to increasing bioavailable isoflavones. [18]. 

Moreover, some LAB, when assumed through the consumption of fermented foods, show antioxidant effects, which differ among species due to different concentrations and types of antioxidants, and therefore could play a pivotal role in human health [19,20].

Among LAB species, *Lactiplantibacillus plantarum* has the Qualified Presumption of Safety (QPS) status from the European Food Safety Authority (EFSA) and the Generally Recognized as Safe (GRAS) status from the US Food and Drug Administration (US FDA) [21]. In addition, this bacterial species has a documented history as a microbial food culture [22,23,24]. Based on previous studies [25], in this work, we used the *Lp. plantarum* LP95 strain which possesses antimicrobial, β-glucosidase activities and exopolysaccharide (EPS) production capacity, as a starter to obtain fermented organic soymilk without the use of stabilizers, thickeners, and acidity regulators. 

Therefore, the main aim of our study was to evaluate the antioxidant capacity of fermented soymilk and its enrichment in isoflavone aglycone.

Furthermore, the fermented soymilk was characterized by its flow behavior and apparent viscosity.

## 2. Materials and Methods

### 2.1. Bacterial Strain 

In this study, *Lp. plantarum* LP95 (Genbank accession number: OM033654), isolated from fermented pollen (bee bread), was cultured at 37 °C in MRS medium (de Man, Rogosa and Sharpe Broth, Oxoid Ltd., Basingstoke, Hampshire, UK). After 12 h, bacterial cells were harvested by centrifugation (10,000 rpm, 5 min, 4 °C) and washed with sterile saline solution (0.9% *w*/*v* NaCl). The supernatant was discarded, and the cell pellet was recovered, resuspended in saline solution and properly diluted to adjust cell concentration before being used as the starter.

### 2.2. Chemicals 

All chemicals were purchased from Sigma (Sigma-Aldrich, Merck KGaA, Darmstadt, Germany). Eluents and water for analytical analysis were purified using a Milli-Q system (Millipore, Burlington, MA, USA).

### 2.3. Preparation of Soymilk 

Handcrafted soymilk was prepared according to Wang et al. [26] with some modification by soaking 400 g of mature yellow organic soybeans (*Glycine max*, marketed by BV&FdL S.r.l., Bentivoglio, Italy) in 1200 mL of sterile distilled water for 12 h. 

Subsequently, soybeans were drained, dried, and blended with 1400 mL of water. The resulting product was subjected to blanching (90 °C, 10 min), afterward filtered and sterilized at 121 °C for 15 min, and finally cooled for the inoculum phase. Next, the entire mass was homogenized by stirring before adding the microbial starter. After the starter inoculation, soymilk was portioned into three different sterile glass containers (200 mL) for subsequent experimental and analytical steps.

### 2.4. Screening for Optimum Temperature

To optimize the fermentation phase, preliminary tests were carried out inoculating the obtained soymilk with *Lp. plantarum* LP95 at a concentration of 10^6^ CFU/mL at three temperatures (20 °C, 28 °C, and 37 °C). The pH and viable cell counts were monitored at 0, 3, 6, 9, 24, 32, 48, and 72 h.

### 2.5. Production of Fermented Soymilk 

According to the preliminary screening for the optimum temperature, the fermentation phase was conducted at 37 °C, inoculating the soymilk with *Lp. plantarum* LP95 (10^8^ CFU/mL). After 24 h, the clot was broken, and the final product was stored at 4 °C. Samples were analyzed immediately and every 7 days until the 49th day.

### 2.6. Determination of Water-Holding Capacity (WHC)

WHC is defined as the capacity of soymilk to retain all or part of its water, and its determination was assayed according to the method described by Li et al. [5]. Briefly, fermented soymilk (30 g) was centrifuged at 8000 rpm at 4 °C for 15 min. The supernatant was collected, weighed, and the *WHC* was calculated using Equation (1):

(1) $$WHC\;\left(\%\right)=\left(1-\frac{W_{1}}{W_{2}}\right)\times 100$$

*W*_1_: weight of supernatant after centrifugation (g); *W*_2_: fermented soymilk weight (g).

### 2.7. Flow Behavior and Apparent Viscosity 

The flow behavior of the fermented soymilk was evaluated by a rotational rheometer (Haake Mars III, Thermo Scientific, Karlsruhe, DE) with a plate–plate measuring system (20 mm in diameter, 1 mm gap distance). Flow curves were generated by varying the shear rate from 0.001 to 300 s^−1^ over 2 min, recording the shear stress and viscosity values at the temperature of 37 °C (during the fermentation step) and 4 °C (during storage). The temperature was controlled by a heating and cooling system (Phoenix II, Thermo Scientific, Karlsruhe, Germany) combined with a Peltier system [27,28]. In addition, the apparent viscosity was obtained from flow curves at a shear rate of 50 s^−1^ at 4 °C [29]. Non-inoculated soymilk was used as the control.

### 2.8. Chemical Analysis

For subsequent chemical analyses, soymilk samples were frozen at −40 °C and then freeze-dried under vacuum at 15 Pa in a freeze-drier Genesis 25 ES dryer (VirTis Genesis 25ES, SP Industries Inc., Gardiner, NY, USA) for 48 h (maximum shelf temperature +20 °C). After freeze-drying, the samples were stored at room temperature. The freeze-drying treatment was necessary to guarantee the stability of the soymilk for the subsequent analytical steps. The moisture content (%) of the freeze-dried soymilk was determined according to the method described by Jung et al. [30].

#### 2.8.1. Determination of Isoflavones 

The content of isoflavones in soymilk during the fermentation stage was determined by HPLC, using a Dionex (Dionex, Sunnyvale, CA, USA) Ultimate 3000 quaternary pump equipped with a degasser, thermostated column compartment, and a diode array detector (DAD). Soymilk was sampled at 0, 3, 6, 9, and 24 h from the beginning of fermentation and subjected to freeze-drying. Then, an aliquot of 500 mg freeze-dried soymilk was subjected to a methanolic extraction, according to the method described by Fahmi et al. [31]. Briefly, 25 μL of methanolic extract was injected into a reversed-phase column (Phenomenex Luna 5 µm C18 100 Å 250 × 4.6 mm) protected with Security Guard Phenomenex (Phenomenex Inc., Torrance, CA, USA). Elution was performed at a flow rate of 1 mL/min at 35 °C, using a solution of phosphoric acid 0.1% (eluent A) and absolute methanol (eluent B), setting the following gradient elution profile expressed as %B: 0 min 28%, 2 min 37%, 30 min 73%. Isoflavones were detected at a wavelength of 254 nm. The identification and quantification were performed using Chromeleon software ver. 6.80 through the retention times and calibration curves of daidzein, daidzin, genistein, genistin, glycitein, and glycitin, as external standards. Results were expressed as mg/100 g dry weight (D.W.).

#### 2.8.2. Antioxidant Activity (ABTS Assay)

The 2,2′-Azino-bis(3-ethylbenzothiazoline-6-sulfonic acid) diammonium salt radical cation (ABTS^•^+) was used for estimating the total antioxidant activity (TAA), according to the method described by Re et al. [32]. Briefly, ABTS was dissolved in water to a 7 mM concentration. ABTS radical cations (ABTS^•^+) were produced by reacting the ABTS methanol solution with 2.45 mM potassium persulfate. The ABTS^•^+ solution was diluted with cold pure methanol up to an optical density (OD) of 0.700 at 745 nm. An aliquot of 20 mg of freeze-dried soymilk sample was subjected to methanolic extraction. After 2 h, 100 μL of methanolic extract was mixed with 900 μL of the ABTS^•^+ solution, and the decrease in absorbance was recorded using a BioSpectrometer (Eppendorf, Hamburg, Germany). Trolox was used as standard for the calibration curve. The TAA in soymilk was assayed at 0, 3, 6, 9, and 24 h after inoculum and expressed as the Trolox equivalent antioxidant capacity (TEAC; mg Trolox Eq./100 g D.W.).

#### 2.8.3. Thiobarbituric Acid Reactive Substance (TBARS) Assay

The TBARS assay was used to estimate lipid peroxidation using malondialdehyde (MDA) as a marker. To 40 mg of freeze-dried soymilk, 1 mL of ethanol/water solution (80:20 *v*/*v*) was added, and the suspension was vigorously shaken. Then, samples were centrifuged at 13,000 rpm, and the supernatant was used to generate the malondialdehyde–thiobarbituric acid (MDA-TBA) complex, according to the method described by Hodges et al. [33], with some modifications. Absorbances were detected at 532, 440, and 600 nm, using a BioSpectrometer (Eppendorf, Hamburg, Germany). An aqueous solution of malondialdehyde bis(dimethyl acetal) was used as a standard for the calibration curve. The quantities of MDA during the fermentation phase were calculated using the modified equation reported by Landi [34].

### 2.9. Statistical Analysis

Statistical analysis was performed by analysis of variance (ANOVA), followed by Tukey’s multiple comparisons, while a *t*-test was used for the analysis of the significance of the apparent viscosity during the fermentation stage, using SPSS Statistics 21 software (IBM Corp., Armonk, NY, USA). The calculations were performed on data obtained from the analysis carried out on three independent aliquots (*n* = 3) obtained from a single initial mass of soymilk after starter microbial inoculation. All data were expressed as a mean ± standard deviation (±SD). 

## 3. Results

### 3.1. Preliminary Fermentation Trial

**Figure 1:**
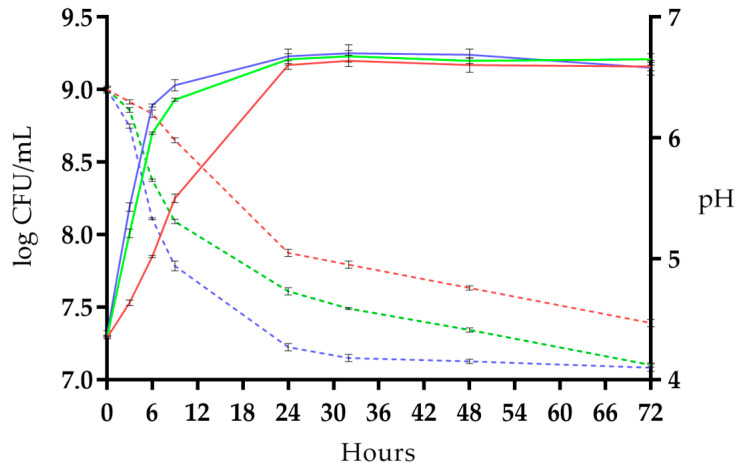
Viable cell count (log CFU/mL) of *Lp. plantarum* LP95 (continuous line) and pH values (dashed line) during the fermentation of soymilk at 20 °C (red line), 28 °C (green line), and 37 °C (blue line). Error bars indicate the standard deviation of the means (*n* = 3).

Although the fermentation kinetics have a similar pattern, preliminary tests revealed, as shown in Figure 1 and Tables S1 and S2 (Supplementary Materials), that 37 °C was the optimal temperature for the fermentation of soymilk using *Lp. plantarum* LP95. In detail, at this temperature, the viable cell counts increased progressively from 7.32 log CFU/mL to a value greater than 9 log CFU/mL, after 9 h of fermentation. Moreover, as shown in Figure 1, after 9 h at 37 °C, the fermented soymilk reached values below pH 5.0, lower than those recorded in tests at 20 °C (pH 5.98) and 28 °C (pH 5.31).

### 3.2. Microbiological and Physicochemical Parameters during Fermentation and Storage 

**Figure 2:**
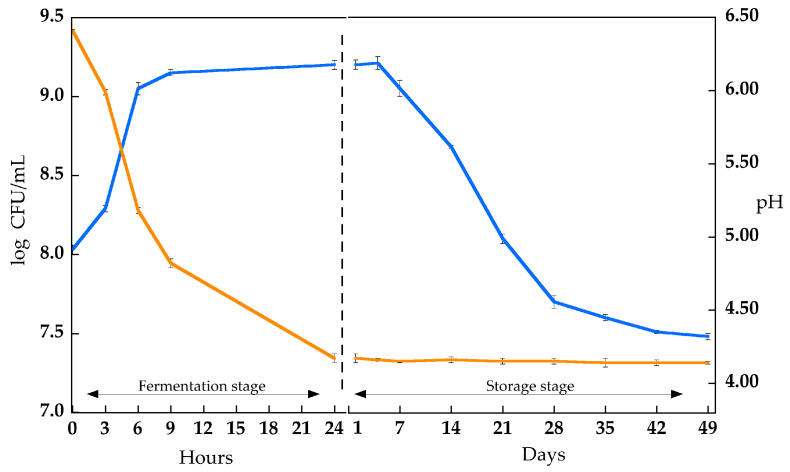
Viable cell count (log CFU/mL) of *Lp. plantarum* LP95 (blue line) and pH values (orange line) during soymilk fermentation (37 °C; 24 h) and fermented soymilk storage (4 °C; 49 days). Error bars indicate the standard deviation of the means (*n* = 3).

Figure 2 shows the viable cell count and pH values using *Lp. plantarum* LP95 in soymilk during the fermentation and storage stages (see also Table S3). As inferred from the viable cell count of *Lp. plantarum* LP95, it reached a value of approximately 9 log CFU/mL during the fermentation phase, which remained constant over the following seven days. After 7 days, a fast decrease in viable cell count occurred, followed by a slower decrease, starting on the 28th day. However, the viable cell count remained above 7 log CFU/mL for up to 49 days of storage. Regarding the pH value of fermented soymilk, an acidification process was evident during fermentation (from 6.5 to 4.2), while during storage, the pH value remained unchanged. In addition, the WHC of fermented soymilk was determined after 24 h, and it reached a value of 43.1 ± 0.3%.

### 3.3. Flow Behavior and Apparent Viscosity of Fermented Soymilk

**Figure 3:**
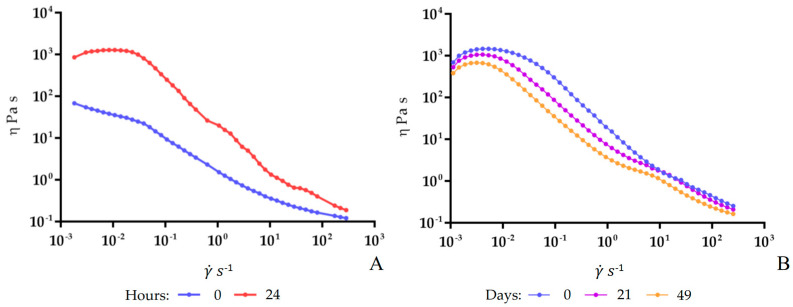
Viscosity curves of soymilk during fermentation using *Lp. plantarum* LP95 as a starter at 37 °C (**A**) and fermented soymilk during storage at 4 °C (**B**) at different times: viscosity (η) as a function of the shear rate ($\dot{\gamma }$).

**Figure 4:**
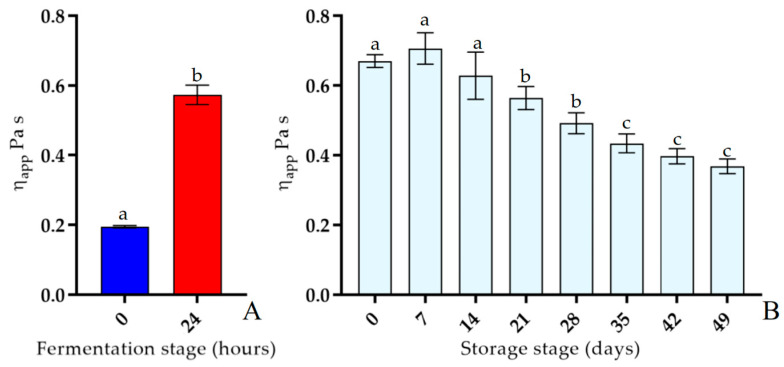
Apparent viscosity at 50 s^−1^ of soymilk during fermentation using *Lp. plantarum* LP95 as a starter at 37 °C (**A**) and fermented soymilk during storage at 4 °C (**B**) at different times. All values are expressed as the mean ± standard deviation (*n* = 3). Different lowercase letters in each bar indicate significant differences *(p* < 0.05).

It is well-known that the correlation between the shear stress (τ) and the shear rate ($\dot{\gamma }$) defines the flow behavior of a sample, which can be classified as Newtonian behavior if the viscosity (η) does not vary with deformation or non-Newtonian behavior when the viscosity varies as a function of the applied shear rate or shear stress. In this study, the apparent viscosity of fermented soymilk was measured to give preliminary rheological information concerning fermented soymilk. The flow behavior of fermented soymilk during both fermentation (37 °C) and storage (4 °C) stages was evaluated, and the obtained curves are depicted in Figure 3A,B, respectively. In detail, all samples showed a non-Newtonian shear-thinning behavior, so the apparent viscosity decreased when the shear rate increased. In Figure 3A, it is possible to observe how fermentation led to changes in the soymilk, with a visible increase in viscosity after 24 h of fermentation. During the storage stage (Figure 3B), a slight decrease in the viscosity of the sample was observed, starting after 21 days. The consistency coefficient (*K*) and flow behavior index (*n*) were obtained as described by Hamet et al. [35], fitting the flow curves with the Ostwald–de Waele model, and the results are reported in Table S4. Moreover, the apparent viscosities of the fermented soymilk at a shear rate of 50 s^−1^ during the fermentation and storage stages are shown in Figure 4A,B, respectively, while the numerical data are reported in Tables S5 and S6. In detail, after 24 h of fermentation, the values of apparent viscosity were different compared to the sample at 0 h. However, after clot rupture at 7 days of storage (4 °C), the highest level of apparent viscosity occurred, while at 21 days, a rapid decline was observed. 

### 3.4. Bioconversion of Isoflavones during Fermentation

Soymilk fermentation, using *Lp. plantarum* LP95 as a starter, significantly increased isoflavones in the aglycone-form. In detail, the results reported in Table 1 showed that the glycosylated isoflavones in soymilk, immediately after the inoculum (0 h), contained daidzin, glycitin, and genistin (11.26 ± 0.27, 39.34 ± 1.00, 40.39 ± 1.27 μmol/100 g D.W.), while the aglycone derivatives were daidzein, glycitein, and genistein (25.64 ± 0.02, 1.58 ± 0.18, and 21.58 ± 0.39 μmol/100 g D.W.). The content of daidzein, glycitein, and genistein increased after 24 h of fermentation, reaching the highest levels of 48.45 ± 1.30, 5.10 ± 0.16, and 56.35 ± 1.02 μmol/100 g D.W., respectively. The moisture content (%) of freeze-dried soymilk was 0.912 ± 0.008.

### 3.5. Changes in Antioxidant Activity during Fermentation

**Figure 5:**
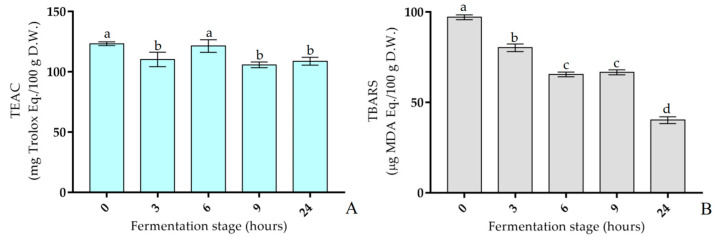
Variations in TEAC expressed as mg Trolox Eq./100 g D.W. (**A**) and TBARS expressed as µg MDA Eq./100 g D.W. (**B**) in soymilk during fermentation using *Lp. plantarum* LP95 as a starter. All values are expressed as the mean ± standard deviation (*n* = 3). Different lowercase letters in each bar indicate significant differences (*p* < 0.05).

During the fermentation stage, the measured values of TAA were significantly different compared to the soymilk immediately after inoculum (0 h). In particular, the results illustrated in Figure 5A and the numerical data reported in Table S7 have shown that the TEAC at 0, 3, 6, 9, and 24 h was 123.35 ± 1.58, 110.30 ± 6.03, 121.42 ± 5.26, 105.79 ± 2.34, and 108.76 ± 3.25 mg Trolox Eq./100 g D.W., respectively. Specifically, the percentage decrease in TEAC was already about 10% at 3 h; at 6 h, it was only 2%, and at 9 and 24 h, it was around 13% compared to the sample at the beginning of fermentation.

### 3.6. Variation in MDA during Fermentation

The evolution of MDA levels during fermentation was assayed. In particular, the detected values showed significant differences during the fermentation stage. As reported in Figure 5B and Table S8, the highest level of MDA concentration was detected at 0 h (97.11 ± 1.33 µg MDA Eq./100 g D.W.); however, it significantly decreased at 3 h (80.26 ± 2.12 µg MDA Eq./100 g D.W.), while between 6 and 9 h (65.53 ± 1.30; 66.68 ± 1.35 µg MDA Eq./100 g D.W., respectively), there were no significant differences between the detected values. Finally, at 24 h, the MDA value reached its lowest level (40.17 ± 1.88 µg MDA Eq./100 g D.W.). 

## 4. Discussion

The formulation of functional foods based on the use of fermentative starters that also possess probiotic properties is becoming an aspect of growing interest in the field of food biotechnology. In this investigation, *Lp. plantarum* LP95 showed good fermentation performance and some properties that overall improved the biofunctional characteristics of unfermented soymilk. It was also inferred that carbohydrates naturally present in soymilk, such as stachyose, raffinose, sucrose, glucose, and fructose, act as substrates for fermentation by LAB, leading to a lowering of pH due to the release of lactic acid [36,37]. The used starter LP95, after 24 h of fermentation at 37 °C, produced a compact clot. After clop rupture, the determined WHC was similar to data obtained by other authors using different LAB strains in soybean-based products [38,39,40].

The observed viscosity changes that occurred during fermentation were also attributable to protein coagulation phenomena [41]. In fact, as described by Xu et al. [42], the protease activity of *Lp. plantarum* might partially hydrolyze soy proteins by lowering soymilk pH. This negatively charges the denatured soy protein groups, leading to progressive coagulation. In addition, the ability to produce EPSs from *Lp. plantarum* LP95 [25] could have a positive influence on the coagulation phase [5]. To clarify this aspect, comparative studies will be conducted in the future on soymilk fermented by *Lp. plantarum* LP95 using non-EPS-producing LAB strains or coagulation with calcium chloride [43,44] as references, with and without the addition of EPSs [45].

The study of the apparent viscosity of samples is important to evaluate how fermentation could affect the sensorial quality of the final product. In general, the properties of fermented products are influenced by different factors, such as the type of microorganisms used and the duration and temperature at which fermentation takes place [46]. 

Samples analyzed in this study possessed a non-Newtonian shear-thinning behavior, where the apparent viscosity decreased when the shear rate increased. This behavior was also observed for other vegetable-fermented types of milk [35,39,47]. The flow behavior of the fermented product was then followed during the refrigerated storage phase. The same behavior observed during fermentation was also found during storage for up to the 14th day, with a drop in viscosity values in the following days. The physicochemical changes could also affect the texture and mouthfeel of the final product, so the apparent viscosity at a shear rate of 50 s^−1^ was obtained, as it can be related to the oral perception during the consumption of semisolid products [48,49]. 

The ability of this *Lactobacillus* to increase the viscosity of soymilk could also be related to the probable presence of EPSs, in agreement with previous studies [35,50,51,52]. In this respect, we can report that during the storage of samples containing fermented soymilk stored at 4 °C, the apparent viscosity remained substantially constant.

The bioavailability of glycosidic isoflavones first requires enzymatic hydrolysis of the sugar moiety, which occurs in the microvillous zone of enterocytes in the intestine [53]. Several LAB strains show specific β-glucosidase enzyme activity that hydrolyzes isoflavone glycosides to aglycones [54]. In detail, the fermentation of soymilk using *Lp. plantarum* LP95 as a starter culture confirmed the complete biotransformation of isoflavone glucosides into their aglyconic forms, which occurred after 6 h during the fermentation. Vegetable polyphenols introduced into a diet with soymilk act as an exogenous antioxidant system. In fact, they have an important antioxidant activity against the negative effects of free radicals, preventing oxidative stress and cell damage [55]. Data obtained on the total antioxidant capacity of the fermented soymilk samples were determined by the ABTS radical scavenging. In the first 3 h of the fermentation process, there was a slight reduction in the antioxidant activity, probably due to a depletion of antioxidant molecules by the bacterial cells used to mitigate oxidative stress [20]. After six hours, there was an increase in the antioxidant activity of the fermented soymilk, probably attributable to the deglycosylation by bacterial bioconversion of isoflavone glycosides. After, the antioxidant activity returns to a decrease, probably due to the scavenging of isoflavonoids [56] produced during bacterial metabolism. 

Furthermore, an important parameter to monitor during food processing is the oxidative state of the final product. The oxidation of lipids, which has been extensively studied, proceeds via a free-radical chain mechanism, giving rise to numerous by-products. The main one of these is MDA, which is, in fact, a marker of oxidative damage in biological systems [57]. Monitoring lipid oxidation is important, as this process leads to the loss of nutritional factors and the development of toxic compounds in food, causing various diseases, such as atherosclerosis [58]. The detection of TBARS is frequently used to determine lipid oxidation. However, as reported by Ghani et al. [59], this measure does not indicate the antioxidant mechanism of action. Despite this potential limitation, there are features of the TBARS test that make it useful as a complement to other widely used screening tests, such as the TAA. In our study, the TBARS assay showed a progressive reduction in MDA levels during the fermentation of soymilk using *Lp. plantarum* LP95. Additionally, this trend coincides with the increase in the amount of detected isoflavone aglycones, and this may suggest a correlation between the two events.

## 5. Conclusions

In this article, we presented a study on the *Lp. plantarum* LP95 strain, which has good potential for improving the quality of fermented soymilk to develop functional food products. The enhancement of the antioxidant activity of soymilk has been shown.

The increase in isoflavone aglycone content during fermentation was a result of β-glucosidase activity toward isoflavone glucosides. This process allowed the obtaining of a soy beverage with enhanced antioxidant capacity able to contribute to the improvement in the health and nutritional status of consumers.

Furthermore, we also report that without using chemical additives, the soymilk obtained and stored at 4 °C has a good shelf life of up to 14 days.

Based on this evidence, *Lp. plantarum* LP95 appears to be a promising candidate as a starter culture to obtain a fermented soymilk rich in isoflavone aglycones and with enhanced health-promoting properties.

To validate this application, future investigations will be conducted on the shelf life and on the organoleptic and nutritional qualities of soymilk fermented by this bacterium.

## Figures and Tables

**Table 1:** Deglycosylation of soymilk isoflavones into aglycones during the fermentation stage of soymilk using *Lp. plantarum* LP95 as a starter. All values are expressed as the mean ± standard deviation (*n* = 3).

Isoflavones (µmol/100 g D.W.)	Fermentation Stage (hours)				
	0	3	6	9	24
Daidzin	11.26 ± 0.27 ^a^	11.08 ± 0.26 ^a^	0.57 ± 0.02 ^b^	0.00 ± 0.00 ^c^	0.00 ± 0.00 ^c^
Glycitin	39.34 ± 1.00 ^a^	37.78 ± 0.55 ^b^	1.45 ± 0.10 ^c^	0.00 ± 0.00 ^d^	0.00 ± 0.00 ^d^
Genistin	40.39 ± 1.27 ^a^	40.11 ± 0.02 ^a^	4.60 ± 0.09 ^b^	0.00 ± 0.00 ^c^	0.00 ± 0.00 ^c^
Daidzein	25.64 ± 0.02 ^b^	25.76 ± 1.11 ^b^	47.71 ± 0.72 ^a^	48.45 ±1.74 ^a^	48.45 ± 1.30 ^a^
Glycitein	1.58 ± 0.18 ^d^	1.65 ± 0.04 ^d^	2.61 ± 0.17 ^c^	3.69 ± 0.01 ^b^	5.10 ± 0.16 ^a^
Genistein	21.58 ± 0.39 ^c^	21.77 ± 0.80 ^c^	51.14 ± 0.84 ^b^	54.75 ± 2.11 ^a^	56.35 ± 1.02 ^a^

Different lowercase letters (a–d) in each row indicate significant differences (*p* < 0.05).

## Data Availability

The data are contained within the article or in the Supplementary Material.

## Supplementary Materials

The following supporting information can be downloaded at https://www.mdpi.com/article/10.3390/antiox12071442/s1, Table S1: Viable cell count (log CFU/mL) of *Lp. plantarum* LP95 during the preliminary fermentation trial of soymilk at 20 °C, 28 °C, and 37 °C, Table S2: Changes in pH during the preliminary fermentation trial of soymilk with *Lp. plantarum* LP95 at 20 °C, 28 °C, and 37 °C, Table S3: Changes in viable cell count (log CFU/mL) and pH in soymilk during the fermentation (37 °C) and storage stage (4 °C) using *Lp. plantarum* LP95 as a starter, Table S4: Values of consistency coefficient (*K*) and flow behavior index (*n*) obtained by fitting the flow curves with the Ostwald–de Waele model, Table S5: Apparent viscosity (Pa s) at 50 s^−1^ of soymilk during the fermentation stage at 37 °C using *Lp. plantarum* LP95 as a starter, Table S6: Apparent viscosity (Pa s) at 50 s^−1^ of fermented soymilk during the storage stage at 4 °C using *Lp. plantarum* LP95 as a starter, Table S7: Variations in total antioxidant activity (TEAC; mg Trolox Eq. 100 g^−1^ D.W.) in soymilk during the fermentation stage using *Lp. plantarum* LP95 as a starter, Table S8: Variations in thiobarbituric acid reactive substance (TBARS; µg MDA Eq. 100 g^−1^ D.W.) in soymilk during the fermentation stage using *Lp. plantarum* LP95 as a starter.
