# Supplementary material for: Antioxidative Properties of Fermented Soymilk Using Lactiplantibacillus plantarum LP95
Source: Antioxidants (Basel). 2023 Jul 18;12(7):1442. doi: 10.3390/antiox12071442 (PMC10376881; doi:10.3390/antiox12071442)
Supplement: Supplementary file 1 [file antioxidants-12-01442-s001.zip › antioxidants-2462353-SI.pdf]

Table S1. Viable cell count (log CFU/mL) of *Lp. plantarum* LP95 during preliminary fermentation trial of soymilk at 20 °C, 28 °C and 37 °C. All values are expressed as mean ± standard deviation (n = 3). Different lowercase letters in each row indicate significant differences ($p < 0.05$).

Fermentation time (hours)	Viable cell count 20 °C	Viable cell count 28 °C	Viable cell count 37 °C
0	7.29±0.01[a]	7.30±0.01[a]	7.32±0.02[a]
3	7.53±0.02[c]	8.01±0.03[b]	8.19±0.03[a]
6	7.85±0.01[c]	8.70±0.01[b]	8.89±0.01[a]
9	8.25±0.03[c]	8.93±0.01[b]	9.03±0.04[a]
24	9.17±0.03[a]	9.21±0.04[a]	9.23±0.05[a]
32	9.20±0.04[a]	9.23±0.04[a]	9.25±0.06[a]
48	9.17±0.05[a]	9.20±0.02[a]	9.24±0.04[a]
72	9.16±0.03[a]	9.21±0.04[a]	9.15±0.05[a]

Table S2. Changes in pH during preliminary fermentation trial of soymilk with *Lp. plantarum* LP95 at 20 °C, 28 °C and 37 °C. All values are expressed as mean ± standard deviation (n = 3). Different lowercase letters in each row indicate significant differences ($p < 0.05$).

Fermentation time (hours)	pH 20 °C	pH 28 °C	pH 37 °C
0	6.40±0.02[a]	6.40±0.03[a]	6.40±0.02[a]
3	6.30±0.02[a]	6.23±0.02[b]	6.10±0.02[c]
6	6.20±0.03[a]	5.65±0.01[b]	5.33±0.01[c]
9	5.98±0.02[a]	5.31±0.02[b]	4.94±0.04[c]
24	5.05±0.03[a]	4.73±0.03[b]	4.27±0.03[c]
32	4.95±0.03[a]	4.59±0.01[b]	4.18±0.03[c]
48	4.76±0.02[a]	4.41±0.02[b]	4.15±0.02[c]
72	4.47±0.03[a]	4.12±0.02[b]	4.10±0.03[b]

Table S3. Changes of viable cell count (log CFU/mL) and pH in soymilk during the fermentation (37 °C) and storage stage (4 °C) using *Lp. plantarum* LP95 as starter. All values are expressed as mean ± standard deviation (n = 3). Different lowercase letters in each column indicate significant differences ($p < 0.05$).

Stages	Time	Viable cell count	pH
Fermentation (hours)	0	8.03±0.03[e]	6.41±0.01[a]
	3	8.29±0.02[d]	5.99±0.02[b]
	6	9.05±0.04[b]	5.18±0.02[c]
	9	9.15±0.02[a]	4.82±0.03[d]
	24	9.20±0.03[a]	4.17±0.03[e]
Storage (days)	1	9.21±0.04[a]	4.16±0.01[e]
	7	9.05±0.05[b]	4.15±0.01[e]
	14	8.68±0.01[c]	4.16±0.02[e]
	21	8.10±0.03[e]	4.15±0.02[e]
	28	7.70±0.04[f]	4.15±0.02[e]
	35	7.60±0.02[g]	4.14±0.03[e]
	42	7.51±0.01[h]	4.14±0.02[e]
	49	7.48±0.02[h]	4.14±0.01[e]

Table S4. Values of consistency coefficient (*K*) and flow behavior index (*n*) obtained by fitting the flow curves with Ostwald de Waele model. All values are expressed as mean ± standard deviation (n = 3). Different lowercase letters in each column indicate significant differences ($p < 0.05$).

	K	*n*	R^2
Fermentation stage (37°C)			
0 hour	0.70±0.04[b]	0.68±0.01[a]	0.992
24 hours	5.27±0.23[a]	0.41±0.01[b]	0.990
Storage stage (4°C)			
0 day	7.40±0.19[a]	0.39±0.01[b]	0.995
21 days	7.18±0.25[a]	0.35±0.01[c]	0.986
49 days	3.36±0.21[b]	0.44±0.01[a]	0.977

Ostwald de Waele model: $\tau = K * \dot{\gamma}^n$

where τ is the shear stress (Pa), $\dot{\gamma}$ the shear rate (1/s), *K* is the consistency coefficient (Pa * s^n), corresponding to the fluid consistency and *n* is the flow behaviour index (dimensionless number).

For *n* values =1 the fluid behaves as a Newtonian fluid, for $n<1$ the fluid is considered pseudoplastic or shear thinning fluid, and for $n>1$ the fluid behaves as dilatant or shear thickening fluid.

Table S5. Apparent viscosity (Pa s) at 50 s^{-1} of soymilk during fermentation stage at 37 °C using *Lp. plantarum* LP95 as starter. All values are expressed as mean ± standard deviation (n = 3). Different lowercase letters in each row indicate significant differences ($p < 0.05$).

Fermentation stage (hours)	
0	24
0.195±0.003[a]	0.573±0.028[b]

Table S6. Apparent viscosity (Pa s) at 50 s^{-1} of fermented soymilk during storage stage at 4 °C using *Lp. plantarum* LP95 as starter. All values are expressed as mean ± standard deviation (n = 3). Different lowercase letters in each row indicate significant differences ($p < 0.05$).

Storage stage 4 °C (days)							
0	7	14	21	28	35	42	49
0.670±0.018[a]	0.706±0.045[a]	0.628±0.068[a]	0.564±0.033[b]	0.492±0.030[b]	0.434±0.027[c]	0.397±0.022[c]	0.368±0.021[c]

Table S7. Variations of Total Antioxidant Activity (TEAC; mg Trolox Eq. 100 g^{-1} D.W.) in soymilk during fermentation stage using *Lp. plantarum* LP95 as starter. All values are expressed as mean ± standard deviation (n = 3). Different lowercase letters in each row indicate significant differences ($p< 0.05$).

Fermentation stage (hours)				
0	3	6	9	24
123.35±1.58[a]	110.30±6.03[b]	121.42±5.26[a]	105.79±2.34[b]	108.76±3.25[b]

Table S8. Variations of Thiobarbituric Acid Reactive-Substances (TBARS; µg MDA Eq. 100 g^{-1} D.W.) in soymilk during fermentation stage using *Lp. plantarum* LP95 as starter. All values are expressed as mean ± standard deviation (n = 3). Different lowercase letters in each row indicate significant differences ($p< 0.05$).

Fermentation stage (hours)				
0	3	6	9	24
97.11±1.33[a]	80.26±2.12[b]	65.53±1.30[c]	66.68±1.35[c]	40.17±1.88[d]
